# Florid dermatopathic lymphadenopathy—A morphological mimic of Langerhans cell histiocytosis

**DOI:** 10.1002/ccr3.1663

**Published:** 2018-06-22

**Authors:** Aishwarya Ravindran, Gaurav Goyal, Jarrett J. Failing, Ronald S. Go, Karen L. Rech

**Affiliations:** ^1^ Department of Laboratory Medicine and Pathology Mayo Clinic Rochester MN USA; ^2^ Division of Hematology Mayo Clinic Rochester MN USA

**Keywords:** dermatopathic lymphadenopathy, Langerhans cell histiocytosis

## Abstract

The histopathology of reactive florid dermatopathic lymphadenopathy shows overlap with Langerhans cell histiocytosis (LCH) involving the lymph node, which may lead to misdiagnosis. These entities can be distinguished by recognition of the sinus‐based distribution of Langerhans cells in LCH, in contrast to the paracortical distribution of Langerhans cells, pigment‐laden histiocytes, and small lymphocytes in dermatopathic lymphadenopathy.

## CASE DESCRIPTION

**Figure 1:**
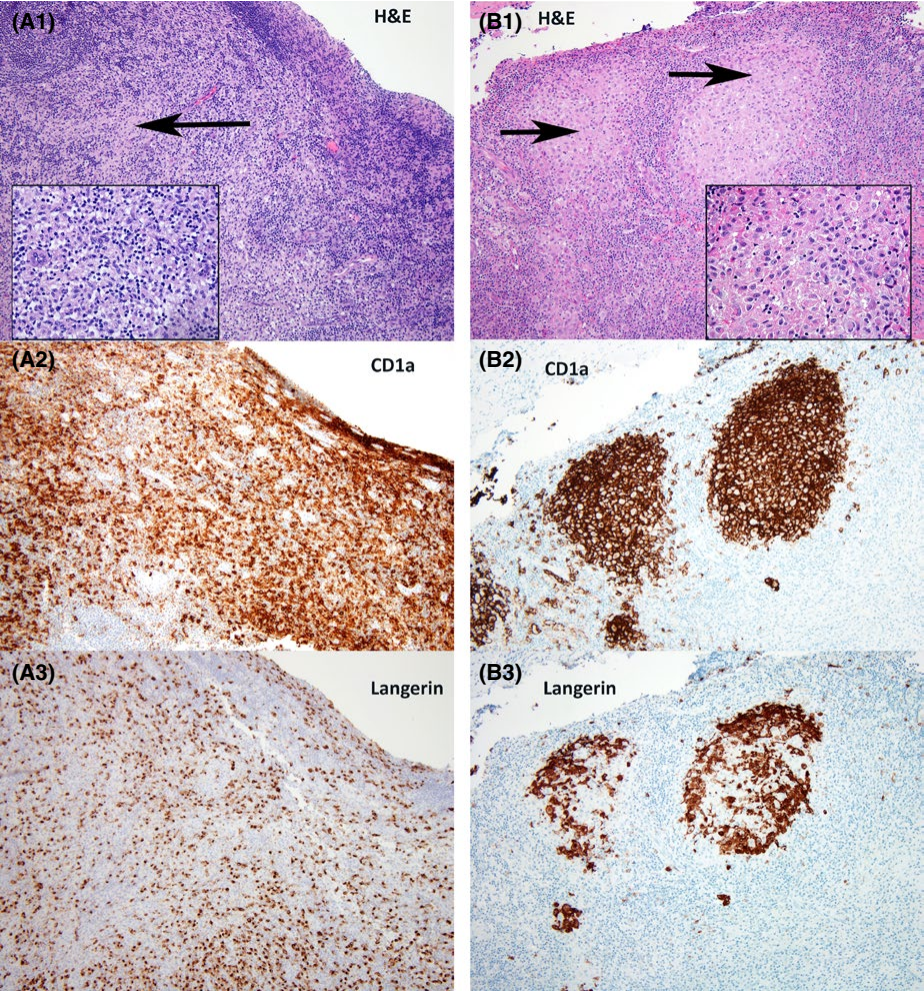
Panel (A) Florid dermatopathic lymphadenopathy is characterized by paracortical expansion by small lymphocytes, histiocytes, and Langerhans cells (A1: H&E—Hematoxylin and Eosin, original magnification—100× and inset—400×).The reactive Langerhans cells express CD1 a (A2‐100×) and Langerin (A3‐100×). Panel (B) In contrast, Langerhans cell histiocytosis shows the infiltration of Langerhans cells restricted to the lymph node sinuses (B1: H&E—Hematoxylin and Eosin, original magnification—100× and inset—400×). The neoplastic Langerhans cells express CD1a (B2‐100×) and Langerin (B3‐100×)

A 21‐year‐old male presented with fever, generalized pruritus and nonblanching petechial and papular skin rash. The rash had a centripetal distribution, initially appearing on the extremities and spreading to the trunk. Laboratory tests revealed normal white blood cell count with mild lymphopenia of 0.8 × 10^9^/L, elevated C‐reactive protein, positive antinuclear antibody and elevated liver function tests (serum alanine transaminase: 277 U/L, serum aspartate transaminase: 81 U/L, alkaline phosphatase: 170 U/L). He was suspected to have Rocky Mountain spotted fever (RMSF) and initiated therapy with doxycycline. However, viral and tick serologies (RMSF, Lyme, Ehrlichia) were negative. He continued to have persistent fever with a significant malaise, and developed bilateral uveitis and a palpable mass in the right chest wall. Imaging studies showed ground glass opacities of right middle and lower lobes of the lungs and moderately fluorodeoxyglucose (FDG)‐avid mediastinal, hilar, and axillary lymph nodes. Eventually, an excisional biopsy of a right chest wall lymph node was performed and he was diagnosed with Langerhans cell histiocytosis (LCH). Flow cytometry showed phenotypically normal T‐cell and B‐cell populations. He was initiated on vinblastine and prednisone for treatment of LCH, and after 3 cycles, he had symptomatic improvement and a decrease in FDG‐avid lesions on imaging studies. Next‐generation sequencing on tissue biopsy did not reveal any mutations, including mitogen‐activated kinases (MAPK) pathway alterations. The patient came to our institution for a second opinion. Histopathological review of his lymph node biopsy showed that the paracortex was expanded by a mixed population of Langerhans cells, pigment‐laden histiocytes, and small lymphocytes (Figure 1, Panel A). Although the presence of numerous Langerhans cells was confirmed with immunohistochemistry for CD1a and Langerin, the morphology and pattern of distribution of these cells pointed away from LCH, since the latter is characterized by a sinus‐based infiltrate of Langerhans cells (Figure 1, Panel B). Based on this, we arrived at a diagnosis of florid dermatopathic lymphadenopathy, a reactive pattern seen in lymph nodes of patients with rashes or other inflammatory skin conditions. The patient continued to improve clinically after discontinuing chemotherapy. This case emphasizes the importance of histopathological re‐evaluation when the clinicopathological correlation is under ambiguity.

## CONFLICT OF INTEREST

None declared.

## AUTHORSHIP

All the authors made substantial contribution to the preparation of this manuscript and approved the final version for submission. AR and KLR: acquired the images. AR, GG, and KLR: drafted the initial version of manuscript. JJF and RSG: revised the manuscript for critically important intellectual content.

